# Performance, Politics and Boris Johnson's Brexit

**DOI:** 10.3389/fpsyg.2021.709756

**Published:** 2021-10-25

**Authors:** Carl Senior, Patrick Stewart, Erik Bucy, Nick Lee

**Affiliations:** ^1^School of Psychology, Aston University, Birmingham, United Kingdom; ^2^Department of Political Science, University of Arkansas, Fayetteville, NC, United States; ^3^College of Media & Communication, Texas Tech University, Lubbock, TX, United States; ^4^Warwick Business School, The University of Warwick, Coventry, United Kingdom

**Keywords:** non-verbal behaviour, politics, performance, populism, Brexit

Modern day politics is a global affair with politicians now having an unparalleled opportunity to communicate manifesto points *via* a range of media (see e.g., Grabe and Bucy, 2009). Most, if not all, significant election campaigns now feature a press officer who directs such broadcasts and regular media training is de rigueur for anybody with political aspirations. Such ubiquitous and uniform treatment of the broadcast message ensures that successful politicians need to develop a unique performance style to stand out from the crowd. Here, we consider the effects that a politician's unique media presence can have on the outcome of an election, specifically the 2019 UK General Election. As will be shown the role of non-verbal (or paralinguistic) communication is key to understanding the efficacy of such a political style.

This election was unique as its outcome gave the winner the legislative authority to enact the UKs eventual withdrawal from Europe and conclude Britain's exit from the European Union (i.e., “Brexit”). The outcome of the 2019 election resulted in a massive gain in power for the eventual winner, Boris Johnson, and the resignation of Jeremy Corbyn's position as leader of the main opposition party, Labour; as well as the complete loss of any parliamentary standing for Joanna Swinson, the leader of the second main opposition party, the Liberal Democrats.

There is no doubt that Brexit featured heavily in the election campaigns of each of the three major political parties at this election. Yet, some argue that the outcome of this election was driven less by a successful “pro-leave” movement that secured the departure and more by a more general national dissatisfaction, in which voters were angry with the drawn-out constitutional processes of the UK government and wanted the Brexit process simply to end (Curtice, 2016; Flinders, 2020a). Indeed, the 2019 election was called to ostensibly solve the constitutional paralysis that emerged in attempting to enact the outcome of the 2016 Brexit referendum.

The outcome of the 2019 election was especially unusual given the fact that at the time public perception of politicians in general was at an historical low (IPSOS MORI, 2020). With the three main political leaders each enjoying less favourable polling than each of their respective counterparts in the previous general elections held 2017 and 2015^1^—it is was clear that politics as usual was not a useful guide.

The national sentiment toward politics in general was also at a historic low at the eve of this election. An audit on political engagement carried out by the Hansard Society in 2019 revealed that nearly half of the sample tested indicated that they felt that they had no influence whatsoever in national decision making with a staggering 75% indicating that they felt that the democratic system in the UK needed to be changed. It was also noted that “*Opinions of the system of governing are at their worst point in 15 years…people are pessimistic about the country's problems and their possible solution, with sizable numbers willing to entertain radical political changes*” (The Hansard Society, 2019, p. 3). A majority of the population indicated that they were currently experiencing “Brexit fatigue” and merely wanted it to go away. Such “Brexistential Angst” (Flinders, 2020b, p. 226) coupled with significant disillusionment in politics provided a perfect opportunity for the successful emergence of a distinctive leader who could develop and deploy a unique performance style to shift the negative attitudes that were universally experienced in the UK, and effect a successful outcome in the election (see also Hay, 2020).

Immediately prior to the 2019 election one could be mistaken for assuming that there was only one political leader in the UK, Boris Johnson, such was his presence in the national media (Yates, 2019 and see also Tolson, 2018). Notwithstanding his own personal levels of popularity (or otherwise), Johnson took advantage of the growing disdain for politics in general and started to develop a unique persona that was replete with symbols that clearly positioned certain politicians (including himself) as unconventional and radical leaders e.g., using the media to generate an “us vs. them” narrative (see e.g., Barr, 2009). This allowed him to generate controversary and apply it to saturate the broadcast media which in turn served to divert debate (e.g., Moffitt, 2016).

From such a perspective, he was effectively demonstrating that *he* was the radical politician who would get things done despite the complexity of parliamentary rules^2^. Such a view is almost diametrically opposed to the common view among political commentators of Johnson as at best something of a bumbler, lurching from one gaffe to another (e.g., Murphy, 2017), or at worst a genuinely bad person (see e.g., Murphy, 2019). The effective communication of such a political position goes beyond linguistic channels and is firmly embedded within the domain of paralinguistic or non-verbal communication.

Corbyn's campaign, on the other hand, was defined by a less aggressive form of communication. Early on the electoral campaign he declared that he would remain neutral in the unlikely event of a second referendum. However, in doing so he also implicitly declared that he was a leader who would continue to abide by the rules that were perceived to have immobilised constitutional debate in the UK, and in turn had led to significant levels of national disillusionment. Further, despite Swinson's Liberal Democrats positioning themselves as the only clearly pro-remain party, she failed to capitalise on the political situation and did not present a viable opposition to Johnson's ubiquitous pro-leave message (Sloman, 2020).

For Johnson such a strategy was very effective as the voting demographic of Brexit was clearly polarised. Working class older voters with few university qualifications voted mostly to leave the EU while middle class and younger university graduates voted to remain (Curtice, 2020). Thus, both Johnson and Corbyn were campaigning to promote policy that was not directly aligned with their traditional electoral bases. However, Corbyn's neutrality merely served to drive dissatisfaction from those who wish to remain in the EU who, without a viable option to Remain, were left to split their vote or support Johnson, who also had the advantage of using Brexit to channel disaffected citizens to vote for him (and indeed a significant number of traditional Labour voters did support him in the ballot box; Curtice, 2020).

Thus, Johnson's approach was clear: use distinct non-verbal behaviour to dominate the media and focus the dissatisfied population with a strong pro-leave message to make inroads into the tradition labour heartlands. As long as Brexit was the key and ubiquitous message in any 2019 general election campaign narrative, there would be no need to promote a policy to target pro-remain voters. Here the threat of Brexit continuing was merely the means by which the Conservatives gained a considerable increase in power in an election that was promoted to a population that was significantly dissatisfied with elections (Macdougall et al., 2020).

Johnson's unique form of communication allowed him to dominate the news narrative while also enabling a focused application of territorial targeting of traditional Labour-held areas outside of Labour's London heartland (Cooper and Cooper, 2020; Cutts et al., 2020). Coupled with the effective application of “Brexit fatigue,” this ensured Johnson could develop a unique media style that also allowed him to separate the Conservative party message from other pro-leave political parties that may have had more of a right-wing narrative (Goodman, 2020). As a unique communication strategy, this was so effective it resulted in what was tantamount to the complete annihilation of political opposition and the domination of the Conservative party in the UK political arena (Cutts et al., 2020).

Indeed, results from the May 2021 local government and Hartlepool by-elections strongly support this narrative, with Conservative support strengthening. This is unprecedented for a government which has been in power now for well over a decade. However, the presence of a large Boris Johnson-shaped blimp over Hartlepool to celebrate his victory does somewhat suggest that normal rules no longer apply. While copious ink has been spilt on commentary regarding the changing electorate, and the failings of Johnson's opponents, the scientific question is perhaps more interesting than the political one; specifically, what (if any) mechanisms did Johnson employ to ensure his form of populism was so effective?

It is clear that under Johnson's superficially clownish behaviour there existed a very serious statesman who was able to communicate non-verbally with great precision. Some may credit this to luck rather than judgement, of course, although we suggest that in the absence of evidence to the contrary, the repeated success of Johnson suggests at least some conscious behavioural and strategic method behind what may appear madness. Indeed, in support of this reading, previous accounts of his unique political style have been termed “Borisonian Populism” (Flinders, 2020a).

We suggest here that Johnson's adroit use of non-verbal channels should also be included under this term. Such non-verbal channels traditionally refer to how behaviours such as facial displays, eye gaze, shifts in posture, body language, and even vocalic utterances etc. are communicated (Giri, 2009). Johnson's dominance of the media, fuelled by his near-constant willingness to court controversy (which are two main elements of his populist performative strategy), would obviously be augmented by an effective repertoire of non-verbal displays. It is clear that Johnson's unique performative style strongly reinforced his verbal message and led to his current domination of the political arena (Cutts et al., 2020)^3^. Future work in this area should consider the central role that non-verbal behaviours play in the effective communication of a particular political strategy (see e.g., Tur et al., 2021).

However, despite the success of Johnson's performance style on the outcomes of the 2019 general election there are a number of questions that still remain to be answered. First, while there is no doubt that Johnson's non-verbal style of communication was indeed highly effective, the question is to what extent is this style directly and consciously transferable to other politicians? In other words, is this a fortunate conflation of natural style with a highly receptive audience? Or is it something more? Similarly, it also remains to be seen whether or not the effect is context specific or is generally effective in other “non-Brexit” related scenarios. Finally, perhaps the most important question is to what extent does a unique non-verbal communication style effect the electorate? More work is needed before the important role of non-verbal channels in driving political outcome with a populist performative style is fully understood.

## Author Contributions

All authors listed have made a substantial, direct and intellectual contribution to the work, and approved it for publication.

## Conflict of Interest

The authors declare that the research was conducted in the absence of any commercial or financial relationships that could be construed as a potential conflict of interest.

## Publisher's Note

All claims expressed in this article are solely those of the authors and do not necessarily represent those of their affiliated organizations, or those of the publisher, the editors and the reviewers. Any product that may be evaluated in this article, or claim that may be made by its manufacturer, is not guaranteed or endorsed by the publisher.

## Footnotes

^1^Indeed, such was the degree of dissatisfaction that Johnson, who scored minus 20% in public satisfaction ratings, was still 2% *more* popular than Swinson and 24% more popular than Corbyn (see Curtice, 2020).

^2^It could be argued that Johnson's proroguing of parliament (an act that was subsequently deemed unlawful by the UK Supreme Court), his refusal to apologise for apparently racist and homophobic behaviour, and his insouciance when faced with the significant criticism for his delay in releasing a report from the Intelligence and Security committee into Russian Interference in the 2016 Referendum, were all examples of astute statecraft that fed directly into the polarisation of the national consensus.

^3^See also Banić (2020) and Müller (2020) for a wider debate as to efficacy of Johnson's non-verbal displays.
